# Progressive Intramuscular Haematoma in a 12-Year-Old Boy: A Case of Acquired Haemophilia A

**DOI:** 10.1155/2018/6208597

**Published:** 2018-10-24

**Authors:** Manori Gamage, Sadeepa Weerasinghe, Mohamed Nasoor, A. M. P. W. Karunarathne, Sashi Praba Abeyrathne

**Affiliations:** ^1^Senior Lecturer, Department of Paediatrics, Faculty of Medical Sciences, University of Sri Jayewardenepura, Nugegoda, Sri Lanka; ^2^Paediatric Registrar, University Paediatric Unit, Colombo South Teaching Hospital Kalubowila, Dehiwala-Mount Lavinia, Sri Lanka; ^3^Senior Registrar, Department of Pathology, Faculty of Medical Sciences, University of Sri Jayewardenepura, Nugegoda, Sri Lanka

## Abstract

Acquired hemophilia A (AHA) is a rare bleeding disorder due to acquired antibodies against coagulation factor VIII (FVIII). It is rare in children less than 16 years old, and the incidence is 0.45/million/year. An otherwise healthy, 12-year-old boy was admitted to the ward with a history of swelling of the right and left forearms, for 1 day duration. He did not have any history of trauma or bleeding disorder. He had prolonged APPTT level with very high antibody titer against factor VIII. His gene expression for factor VIII was found to be normal. He was managed with FEIBA and recombinant FVII activated complexes and prednisolone 1 m/kg/day regime to control bleeding. AHA is associated with several underlying pathologies such as pregnancy, autoimmune diseases, malignancy, medications and infections; however, up to 50% of reported cases are idiopathic. In contrast to congenital haemophilia A, in which haemarthrosis is the hallmark clinical presentation, patients with AHA mainly bleed in to the skin, muscles, and soft tissues. High mortality rate of more than 20% is either to retroperitoneal or intracranial bleeds. Diagnosis is confirmed on isolated prolongation of activated partial thromboplastin time which does not normalize after addition of normal plasma, reducing the factor VIII levels with evidence of FVIII inhibitor activity. They have normal prothrombin time and platelet functions. Management of AHA involves two aspects, namely, eradication of antibodies and maintaining effective haemostasis during a bleeding episode.

## 1. Introduction

Acquired haemophilia A (AHA) is a rare bleeding disorder. It occurs due to acquired antibodies against coagulation factor VIII (FVIII). Generally, it has an incidence rate of 1 to 4 per million/year with a biphasic distribution pattern which demonstrates a small peak in young individuals aged between 20 and 30 years and major peak in individuals around 60–80 years [1]. It is rarer in children less than 16 years, and the incidence is 0.45/million/year [2]. Although this is a rare disorder, it is associated with significant morbidity and mortality.

Although AHA is associated with autoimmune disorders, infections, malignancies, drugs, and pregnancy, no cause was identified in 50% of cases [3].

Here, we report a case of an acquired haemophila A in a previously healthy 12-year-old boy.

## 2. Case Report

Otherwise healthy, 12-year-old boy was admitted to the ward with a history of swelling of the right and left forearms, for 1 day duration.

He gave a history of a swelling in the right forearm first noticed six weeks prior to the current presentation, and it has resolved gradually without any acute intervention. During the initial presentation, the mother claimed that he was treated with a course of amoxycilline for an upper respiratory tract infection prior to the onset of the swelling. Since then, he was well till this current admission.

During this presentation, the swelling of the right elbow joint along with the forearm swelling worsened progressively. He did not have any history of trauma or febrile illness associated with the current presentation.

He denied any bleeding tendency in the past except a history of mild extra bleeding which settled spontaneously following a dental extraction one month back. There was no history of photosensitive skin rashes, renal problems, recent weight loss, or poor appetite. He did not have any family history bleeding disorders.

On examination, he was alert, pale but not icteric or febrile. His weight : height ratio lied between 1 SD and median. He did not have lymphadenopathy, hepatosplenomegaly, or ballotable masses.

Examination of the upper limbs revealed that the range of movements was reduced due to the pain and there was diffused tense swelling of both forearms. But there were no inflammatory changes noted on the over line skin or adjacent joints of the swollen areas. Rest of his systemic examination was unremarkable.

During initial investigations, his full blood count revealed a white cell count of 8.62 × 10^9^ with a normal differential count. His haemoglobin was 7.7 g/dl with a platelet count of 278 × 10^9^/L.

His clotting profile showed a normal PT/INR with normal bleeding and clotting time but his APPT was significantly prolonged (patient: 109.9 seconds; control: 28 secs). His factor assay showed that factor VIII level was <5%, and factor IX level was normal.

His ESR was 25 mm/1^st^ hour, and rest of the investigations including liver function test, 2 D echocardiography, chest X-ray, ultrasound scan of the chest and abdomen, thyroid, profile and LDH level were normal. Antinuclear and antiphospholipid antibodies were negative.

Blood picture was interpreted as iron deficiency anaemia with evidence of active bleeding. Ultrasound of the upper limbs showed that there is a possibility of bleeding into the left forearm muscle with small collection of fluid in the right elbow joint.

As there was prolonged APTT with low factor VII levels and the forearm swelling was progressive, it was decided to treat this patient as haemphilia A, and factor VIII concentration was commenced to achieve a correction of 50%. In spite of commencing the factor correction regime, it was noted that swelling was progressive with significant pain. His haemoglobin dropped significantly indicating active bleeding. Hence, an inhibitor screening was performed (Bethesda assay), and it was 33.6 BU, which is a very significant inhibitor titer. A diagnosis of acquired haemophilia A due to inhibitors was made.

With this high inhibitor titer, it was decided to manage him with the Factor Eight Inhibitor Bypassing Activity (FEIBA) (aPCC-activated prothrombin complex concentrate) 75 U/kg every 12 hours, but his response was unsatisfactory. As a result, he was commenced on recombinant activated factor VII (rFVIIa) 90 micro gram/kg with a marginally satisfactory response. Hence, in addition to this regime, the patient was commenced on prednisolone 1 mg/kg/day to eradicate the acquired antibody response and showed a promising response. He required 3 units of pack cell transfusions during this period.

Currently, he is on prednisolone 10 mg daily, and his recent APTT was 51.2 seconds with an inhibitor level of 1.98 BU.

## 3. Discussion

AHA is a rare bleeding disorder due to auto antibody formation against factor VIII. It was first described in 1940 [4]. Although this is a rare hemorrhagic disorder, it is the most frequent acquired clotting factor disorder [1]. This is found be more frequent among elderly and rare among children less than 16 years of age [2]. The incidence in men and women is similar but more female cases are reported in the younger age between 20 and 30 years as pregnancy is a known predisposing factor for AHA [5].

These antibodies are usually poly clonal immunoglobulins [1] and are also known as inhibitors. AHA is associated with several underlying pathologies such as pregnancy, autoimmune diseases, malignancy, medications, and infections; however, up to 50% of reported cases are idiopathic [1, 3]. In AHA, clinical picture may range from mild or no bleeding to life threating bleeding [3].

In contrast to congenital haemophilia A, in which haemarthrosis is the hallmark clinical presentation, patients with AHA mainly bleed in to the skin, muscles, and soft tissues [1]. AHA is associated with a high mortality rate of more than 20% which is due to either retroperitoneal or intracranial bleeding [3].

**Figure 1:**
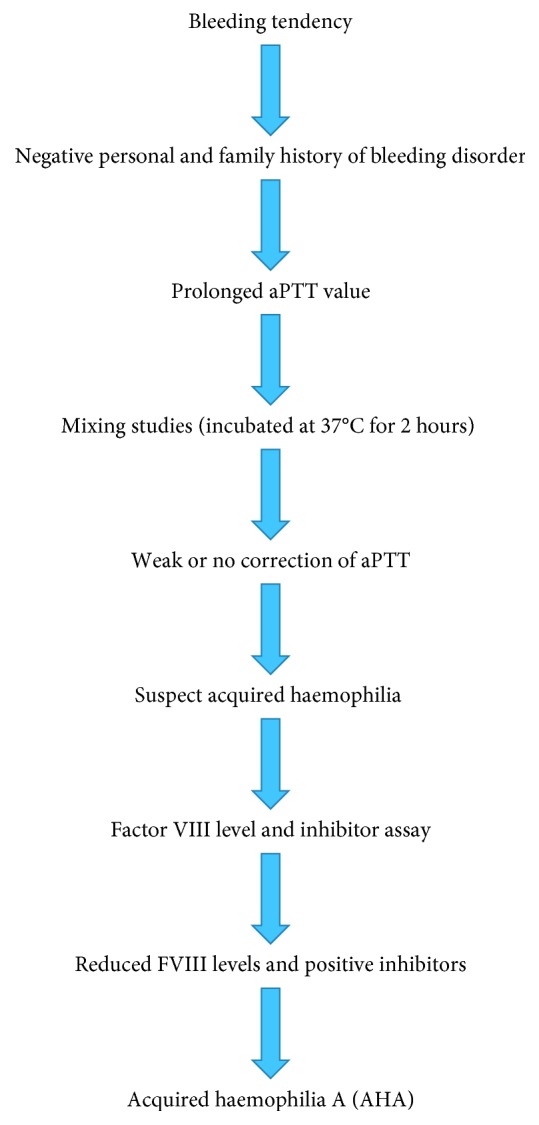
Diagnosis of acquired haemophilia.

Diagnosis of AHA should be suspected in patients present with acute onset of significant bleeding manifestations without a previous history of bleeding disorder [3]. The diagnosis can be confirmed by isolated prolongation of activated partial thromboplastin time (aPTT), which does not normalize after addition of normal plasma and incubation for one two hours, reducing the factor VIII levels with evidence of FVIII inhibitor activity. They have normal prothrombin time and platelet functions [6]. The FVIII inhibitor level is measured by the Bethesda assay and expressed as Bethesda units (BU) [3] (Figure 1).

Management of AHA involves two aspects, namely; eradication of antibodies and maintaining effective haemostasis during a bleeding episode [1, 7].

The options used to control an acute bleed are based on the level of antibody titer [1]. If the patient's antibody titer is low (<5 BU), it can be managed with human factor VIII concentration ± DDAVP administration [1, 7]. If the patient's antibody titer is high (>5% BU), it is managed with bypassing agents. They include recombinant activated factor VII (rFVIIa) and activated prothrombin complex concentrates (aPCC) with an efficacy of 95% and 86% respectively [1].

Our patient was initially treated with aPCC with a poor response, and acute bleeding was controlled with recombinant activated factor VII.

Patients with AHA should be immediately started on immunosuppressive therapy on diagnosis to eradicate FVIII inhibitors [2, 3]. Most frequently used therapeutic agents are corticosteroids: prednisolone (1 mg/kg/day for 3 weeks) as a single agent or in combination with cyclophosphamide (2 mg/kg/day) [2, 3]. A meta-analysis has concluded that cyclophosphamide was superior to prednisolone to eradicate inhibitors but not in overall survival [1]. Our patient was commenced on prednisolone 1 mg/kg/day to eradicate inhibitors.

There is necessary evidence on the effectiveness of other treatment approaches such as immune tolerance regimens and rituximab, when first-line immunosuppressive therapy fails or is contraindicated. But there is conflicting evidence in relation to high dose immunoglobulin therapy [1, 3].

Complete inhibitor eradication is defined as undetectable inhibitor and normal FVIII levels. Patients who are on immunosuppressive therapy need regular follow-up as an out-patient basis for a minimum period of 2 years. Their assessment should include physical examination, full blood count, aPTT, and FVIII inhibitor titer assay [3].

Though AHA is a rare entity, especially in children, it should not be missed in differential diagnosis, provided the clinical presentation is suggestive.
